# Higher risk of gastrointestinal parasite infection at lower elevation suggests possible constraints in the distributional niche of Alpine marmots

**DOI:** 10.1371/journal.pone.0182477

**Published:** 2017-08-01

**Authors:** Stefania Zanet, Giacomo Miglio, Caterina Ferrari, Bruno Bassano, Ezio Ferroglio, Achaz von Hardenberg

**Affiliations:** 1 Department of Veterinary Sciences, University of Turin, Grugliasco (To), Italy; 2 Alpine Wildlife Research Centre, Gran Paradiso National Park, Valsavarenche (Aosta), Italy; 3 Conservation Biology Research Group, Department of Biological Sciences, University of Chester, Chester, United Kingdom; University of Pretoria, SOUTH AFRICA

## Abstract

Alpine marmots *Marmota marmota* occupy a narrow altitudinal niche within high elevation alpine environments. For animals living at such high elevations where resources are limited, parasitism represents a potential major cost in life history. Using occupancy models, we tested if marmots living at higher elevation have a reduced risk of being infected with gastro-intestinal helminths, possibly compensating the lower availability of resources (shorter feeding season, longer snow cover and lower temperature) than marmots inhabiting lower elevations. Detection probability of eggs and oncospheres of two gastro-intestinal helminthic parasites, *Ascaris laevis* and *Ctenotaenia* marmotae, sampled in marmot feces, was used as a proxy of parasite abundance. As predicted, the models showed a negative relationship between elevation and parasite detectability (i.e. abundance) for both species, while there appeared to be a negative effect of solar radiance only for *C*. *marmotae*. Site-occupancy models are used here for the first time to model the constrains of gastrointestinal parasitism on a wild species and the relationship existing between endoparasites and environmental factors in a population of free-living animals. The results of this study suggest the future use of site-occupancy models as a viable tool to account for parasite imperfect detection in eco-parasitological studies, and give useful insights to further investigate the hypothesis of the contribution of parasite infection in constraining the altitudinal niche of Alpine marmots.

## Introduction

Parasite infections are costly to their hosts: the development and maintenance of antiparasitic defenses competes for resources with other life-history functions, such as reproduction, growth, and development of secondary sexual traits [1,2,3,4]. High ectoparasite loads in yellow-bellied marmots *Marmota flaviventer* were related to slower growth, lower overwinter survival, and reduced reproduction, suggesting that ectoparasites are a direct fitness cost for marmots [5] and can lead to indirect density-mediated demographics costs [6]. Natural selection should therefore have favored the evolution of behavioral and life history strategies which minimize the risk of infection. For example, it has been shown that Alpine ibex (*Capra ibex*) actively avoids foraging near feces, possibly as an antiparasitic strategy [7], and Yellow baboons (*Papio cynocephalus*) alternate sleeping groves every few nights to avoid the accumulation of infective parasite ova and larvae [8]. Theory predicts that the cost of maintaining these strategies is traded off by the cost of controlling parasite infection [2, 9]. While there are many studies and examples of tradeoffs between behavioral and life history traits and parasite resistance in wild populations [9, 10, 11], only few studies have investigated the potential role such a tradeoff could have in constraining the distributional niche of wildlife populations to areas in which the cost of the risk of parasite infection is below the cost of maintaining antiparasitic strategies [12, 13, 14].

Wildlife populations occupying narrow altitudinal niches in high elevation environments, such as Alpine marmots (*Marmota marmota*), are potentially a good target species to investigate this hypothesis. Indeed, the constraints of a shorter feeding season, longer snow cover and lower temperatures suffered by marmots at higher elevations, may be compensated by lower parasite loads in situations of comparable predation pressure and population density. Alpine marmots are ground-dwelling sciurids adapted to live at high elevation, where they inhabit meadows and pastures between 1,700 and 2,800 m a.s.l. [15]. Habitat preferred by marmots are characterized by cold and highly seasonal climate, but other factors have been shown to strongly influence the presence of marmots and particularly the availability and quality of food resources and the permanence of snow cover [16,17].

Alpine marmots are territorial [18] and live in family groups [19] which include a resident pair, young of the year and subordinates (young of previous years with delayed dispersal) [20, 21]. Alpine marmots cope with long winter season with stressful ambient temperatures and with the lack of food resources, through hibernation [22] making this the pivotal point of their annual cycle [23, 24, 25]. During the six months of hibernation, Alpine marmots rely exclusively on body fat reserves to fuel all their energetic demands and active summer months are thus of paramount importance to accumulate the necessary resources to overwinter [22]. During hibernation, the mass of the gastrointestinal tract is selectively reduced to decrease energy demands [26]. The parasitic load of adult gastrointestinal helminths is largely reduced through a process known as “self-cure” caused by mucosal atrophy and lumen obliteration [27]. *Ctenotaenia marmotae* and *Ascaris laevis* together with *Citellina alpina* and *Coccidia* of the genus *Eimeria*, typically parasitize Alpine marmots with high prevalences and abundances [24, 28, 29]. The high adaptation of Alpine marmots to their Alpine environment, corresponds to an extreme specialization of their parasites which adapted to the host peculiar life-cycle and developed specific yet different overwintering strategies [27]: from co-hibernation (*C*. *alpina*) [30], to exploitation of Oribatid mites as intermediate hosts (*C*. *marmotae*) [31], to passive resistance in the external environment with larvated eggs (*A*. *laevis)* [32, 33]. Eggs and, oncospheres of these gastrointestinal parasites are excreted in the feces of their hosts [24] and therefore the analysis of fecal samples collected from marmot families along an elevational range could potentially be used to calculate the probability of infection at different elevations. The absence of parasite eggs or oncospheres in a fecal sample is however not a valid diagnostic indicator for the absence of parasitic infection, as it is well known that parasite detection in fecal samples suffers a high rate of false negatives due to their inconstant shedding [34]. This problem is conceptually similar to the problem encountered by ecologists when estimating the probability of a site being occupied by a species from presence-absence data [35]. This kind of data could be modeled with a classic logistic regression, including variables such as elevation as predictors. However, the resulting probability estimate represents a mix of both occupancy (*ψ*) and detection probability (the probability *p* that a species is detected when a site is occupied), providing potentially biased and misleading results [35, 36]. Site-occupancy models [37] have been proposed as a formal solution to this problem. Through a sampling protocol that uses multiple, independent visits to M sampling sites, site-occupancy models effectively disentangle and provide separate estimates of *ψ* and *p*. The response to each visit, is a binary value Y_*ij*_ (detection Y = 1, or non-detection Y = 0) during *j* = 1, …., *i* visits to the *i*^th^ site within the same season [37, 38]. By assuming that the probability of detecting a species when it is present at a given site is mostly influenced by local abundance of the species itself, detection probability *p* becomes an indirect estimator of abundance [39]. Similarly, we can assume that the probability of detection of digestive parasites stages from feces, increases with the actual abundance of these parasites [40]

Here we investigate the hypothesis that Alpine marmots suffer a higher risk of being infected by the gastrointestinal helminths *C*. *marmotae* and *A*. *laevis* at the lower boundary of their elevational range, applying, for the first time in the eco-parasitological context, site occupancy models [37] to the detection/non-detection of eggs and oncospheres recorded in fecal samples repeatedly collected over one active season (April–September 2012) from 22 Alpine marmot territories along the whole elevational range of the species in the Gran Paradiso National Park (Northwestern Italian Alps).

## Material and methods

### Collection of Alpine marmot fecal samples

The study area was located in Valsavarenche, within the Gran Paradiso National Park, Northwestern Italian Alps (45°34’N-7°11’E) spanning the entire elevational range of Alpine marmots [41], from approx. 1700 to 2800 m a.s.l. (Tab.1). A total of 22 sites located within the home range of 22 marmot families previously identified [42] were sampled once every 15 days (±3 days) from mid-April to the end of September 2012. The sampling started as soon as marmots emerged from hibernation and ended when the animals burrowed in autumn; in our study area emergence from hibernation starts around mid-March at low elevation (1700–2000 m a.s.l.) up to the end of April at higher elevation (2000 m a.s.l.and above), according to differences in temperature and exposure (C. Ferrari, *pers*. *obs*.). In sites 6, 7 and 8 sampling had to be interrupted earlier (mid-July 2012) because the unexpected presence of tall grass and of high densities of coprophagous beetles which prevented the detection of viable fecal samples. The sampling covered an overall period of 180 days. Only fresh fecal samples were collected: by direct observation of defecating marmots or from latrines [19], which were available and regularly used by all studied families. Ethical approval for the Alpine Marmot Research Project was obtained by the Gran Paradiso National Park Agency from the Italian National Institute for Environmental Protection and Research (ISPRA). We collected only feces of adults and yearlings and systematically excluded those samples that for shape and dimension could be attributed to pups (< 1 year of age). A total of 605 fecal samples were collected for analysis. All fecal specimens were placed into polythene bags and kept at +4°C until analysis for a maximum period of 5 days.

### Coprological analysis

Each specimen was examined to determine the presence of parasite eggs by the Modified McMaster Method [43] using zinc sulfate (ZnSO_4_·7H2O 330 g, H_2_O brought to 1000 ml, with a density of 1.200) as floatation solution. Fecal samples were diluted 1:15 in the flotation solution (2 g of feces in 28 ml of zinc sulfate—ZnSO_4_). Presence of *A*. *laevis* and *C*. *marmotae* together with those of any other parasite were recorded. Parasitic forms were microscopically identified following the morphological criteria of shape, size and color as specified by Bassano et al. [24], Callait and Gauthier [27] and Babero [33]. Coprological prevalence (P) was calculated as number of samples in which at least one parasite egg/onchosphere was detected, on the total number of samples collected per time period. The Confidence Intervals (CI) for each coprological prevalence value were calculated with a 95% confidence level using the software Epinfo 7 [44].

### Site-occupancy models

Data was collected to be suitable for a site-occupancy model [37] to estimate the probability of presence at each sampling site (*ψ* = occupancy) and relative abundance (*p* = detection probability) of life stages of the two selected parasites *C*. *marmotae* and *A*. *laevis* in the feces collected within the territories of the 22 family groups of Alpine marmots considered in this study. Each territory was directly georeferenced (at the entrance of the main borrow) using a GPS receiver (mean distance between the sampled burrows d = 2343 m). Sampling sites were characterized by two site-specific covariates, namely elevation (meter a.s.l.) and mean yearly solar radiance (kW/h/year) in a Geographic Information System (GIS) environment using the software QGIS 2.8.0 [45] (Table 1). Progressive date of sampling (Julian date) was the only sampling-specific covariate that was included in the model. Occupancy and detection probability were modeled using binomial detection/non-detection data for both *C*. *marmotae* and *A*. *laevis* in the feces collected at each site. Different fecal samples collected in each site where treated in the model as replicated samples, in order to estimate detection probability. Occupancy models were computed using the package ‘Unmarked’ [38] in the statistical environment R version 3.0.2 [46]. The Akaike information criterion (AIC) [47] was used for model selection considering models within a ΔAIC of 2 as equivalent [48]. The survey was conceived to meet the specific requirements of occupancy models: independence between replicated surveys, close population and abundance-induced heterogeneity of *p* [37].

**Table 1:** Number of samples and site-specific covariates at each sampling site.

Site	Number of samples	Elevation (m a.s.l.)	Mean annual Solar radiance (kW/h)
1	2	1748	1843
2	20	1760	1786
3	37	1675	1548
4	25	1683	1530
5	44	1682	1714
6	17	1981	1921
7	7	2004	1778
8	23	2027	1892
9	32	2169	1671
10	46	2180	1704
11	21	2210	1312
12	19	2235	1593
13	42	2301	1809
14	37	2342	1890
15	30	2345	1832
16	34	2551	2003
17	33	2541	2187
18	38	2583	1863
19	42	2831	1588
20	13	2670	1935
21	15	2737	1711
22	10	2640	1291

Elevation (meters a.s.l.) and mean annual solar radiance (kW/h) were considered in the occupancy model as site specific covariates. Both parameters were calculated for each of the 22 marmot families included in the study. The value of each covariate is reported in the table together with the number of samples collected/analyzed from each sampling site.

## Results

### Coprological analysis

Eighteen samples had to be excluded from the study due to insufficient quantity or to unsuitable conservation status (total number of viable specimens n = 587). Oncospheres and/or proglottids of *C*. *marmotae* were detected in 274/587 specimens (P = 46.68%, 95% CI = 42.68–50.72%) from all families and all study sites. *C*. *marmotae* was first detected at the end of May (May 22^nd^), in five fecal specimens from the sampling sites located at the lowest elevation (sites 2, 3 and 5). The highest coprological prevalence was recorded in July, reaching P = 61.90% (95% CI = 53.19%-69.91%) (Table 2) but oncospheres were detected until the end of the sampling period (September 27^th^) within the entire elevational range (sites from 1 to 22).

**Table 2:** Copromicroscopic analysis for *A*. *laevis* and *C*. *marmotae*.

*C*. *marmotae*	April	May	June	July	August	September	Total
absent	11	100	49	48	50	55	313
present	0	5	60	78	65	66	274
**Total**	11	105	109	126	115	121	587
**Coprological Prevalence**	**0.00**	**0.048**	**0.551**	**0.619**	**0.565**	**0.546**	**0.467**
**CI 95%**	0.00–0.269	0.021–0.107	0.457–0.641	0.532–0.699	0.474–0.652	0.457–0.632	0.427–0.507
***A*. *laevis***							
absent	11	105	98	90	37	25	366
present	0	0	11	36	78	96	221
**Total**	11	105	109	126	115	121	587
**Coprological Prevalence**	**0.000**	**0.000**	**0.101**	**0.286**	**0.678**	**0.793**	**0.377**
**CI 95%**	0.00–0.259	0.00–0.035	0.057–0.172	0.214–0.37	0.588–0.757	0.713–0.856	0.338–0.416

The number of samples respectively positive and negative for *C*. *marmotae* and *A*. *laevis* are reported for each month of sampling (columns “April” to “September”) and for the entire study period (column “Total”) together with monthly and total coprological prevalence values and confidence intervals (CI95%).

*A*. *laevis* was first detected in mid-June (June 13^th^) from two families of the lower elevational zone (sites 1 and 2). The overall coprological prevalence across the entire active season and across all sampling sites was P = 37.65% (95% CI = 33.82%-41.64%). Monthly coprological prevalence increased, as the season proceeded, from 10.09% (CI 95% 5.73%-17.17%) in June, to 79.34% (CI 95% 71.28%-85.60%) in September (Table 2). *A*. *laevis* was detected within all families, from all sampling sites excluding those where the sampling had to be interrupted in mid-July (sites 6, 7 and 8) and from where a lower number of samples was collected (sites 1 and 22).

The only other parasite species detected were Coccidia of the genus *Eimeria*.

### Occupancy models

**Fig 1:**
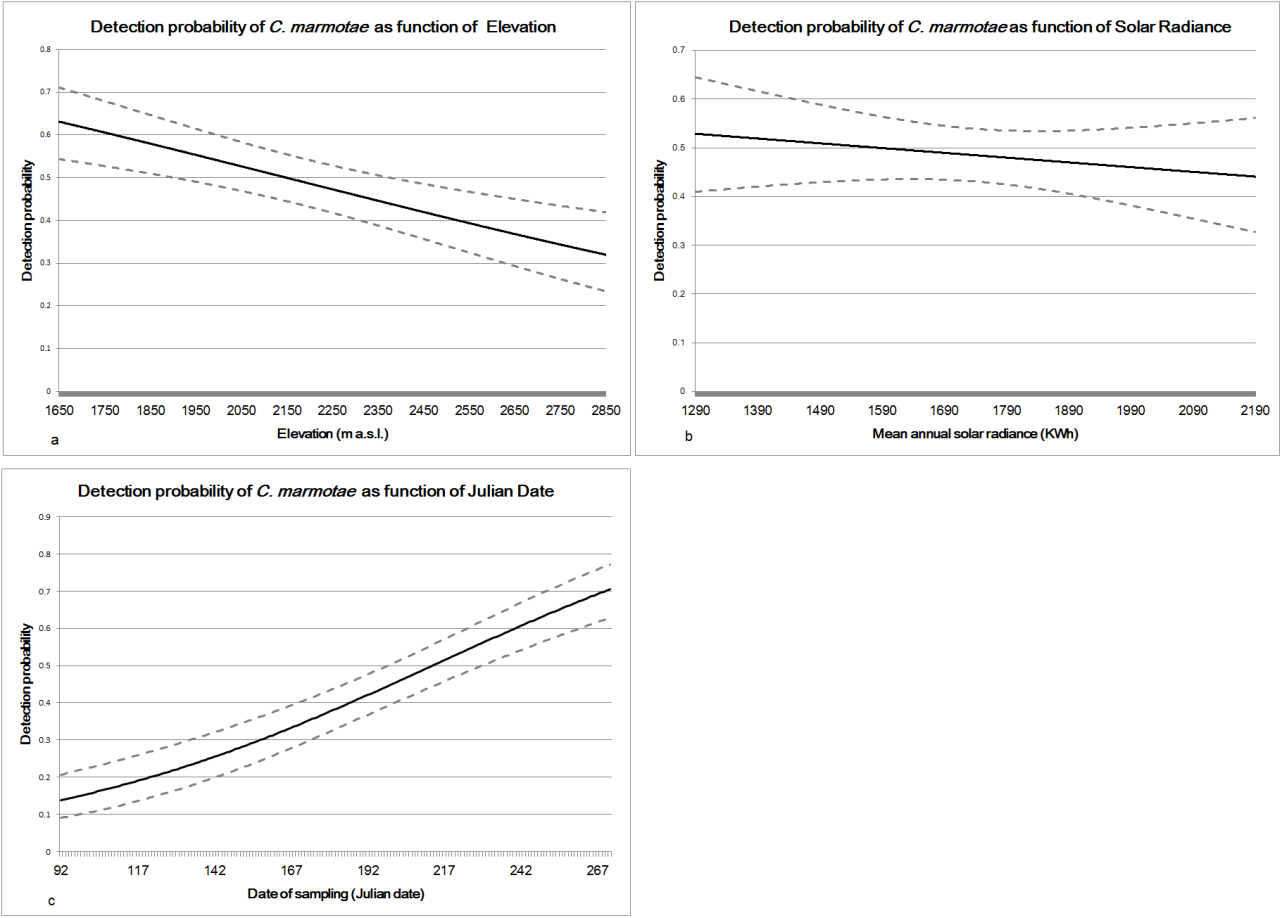
Covariate trend in site occupancy models for *C*. *marmotae*. Detection probability (solid line) and confidence intervals (dashed line) of *C*. *marmotae* are pictured as function of site specific covariates: (1a) elevation (1b) solar radiance and Julian date (1c). The represented values of *p* were obtained considering the median value of each covariate within the model [*ψ(*.*)p(Elev+Rad+Date)*].

*C*. *marmotae* was present in all sampling sites (*ψ* = 1). Site-specific and sampling-specific covariates were added to the constant model *ψ(*.*)p(*.*)* and combined in different models whose performances were ranked by AIC values. The best performing model (AIC = 739.17; Table 3) includes a constant occupancy and linear additive effects of solar radiance, elevation and sampling date on detection probability [Model *ψ(*.*)p(Elev+Date+Rad)*] (Table 4). In the final model both Elevation (Fig 1A) and solar radiance (Fig 1B) are negatively related to *p*, while sampling Date shows a positive relationship with detection probability (Fig 1C).

**Table 3:** AIC-based model selection.

*C*. *marmotae*	AIC	AICwt	cumltvWt	*A*. *laevis*	AIC	AICwt	cumultvWt
ψ(.)p(Elev+Date+Rad)	739.17	8.5e-01	0.85	ψ(.)p(Elev+Date)	471.35	9.1e-01	0.91
ψ(.)p(Elev+Date)	742.56	1.5e-01	1.00	ψ(.)p(Elev+Date+Rad)	476.08	8.6e-02	1.00
ψ(.)p(Date+Rad)	757.89	7.3e-05	1.00	ψ(.)p(Date)	485.61	7.3e-04	1.00
ψ(.)p(Date)	761.10	1.5e-05	1.00	ψ(.)p(Date+Rad)	485.66	7.1e-04	1.00
ψ(.)p(Rad)	797.37	1.9e-13	1.00	ψ(.)p(.)	727.38	2.3e-56	1.00
ψ(.)p(.)	815.16	2.7e-17	1.00	ψ(.)p(Rad)	737.04	1.8e-58	1.00
ψ(.)p(Elev)	821.02	1.4e-18	1.00	ψ(.)p(Alt)	737.97	1.2e-58	1.00
ψ(.)p(Elev+Rad)	847.61	2.4e-24	1.00	ψ(.)p(Elev+Rad)	739.51	5.4e-59	1.00

For each model (null model or models with covariates Elevation (Elev), Solar Radiance (Rad), Julian Date (Date)) the AIC value, AIC weight (AICwt), and cumulative AIC weight (cumltvWt) are reported.

**Table 4:** Details of estimates of detection probability of *C*. *marmotae* and *A*. *laevis*.

***C*. *marmotae***	***Estimate (β)***	**SE**	**z**	**P-value**
(Intercept)	1.251	0.458	2.733	0.006
Elev	-0.001	0.000280	-3.852	0.0001
Date	0.015	0.002	7.586	< 0.0001
Rad	-0.0004	0.0005	-0.804	0.422
***A*. *laevis***	***Estimate (β)***	**SE**	**z**	**P-value**
(Intercept)	-2.465	0.771	-3.20	0.001
Elev	-0.002	0.0002	-5.28	< 0.0001
Date	0.046	0.004	13.12	< 0.0001

Estimated slopes, standard error (SE), z and p-values for each variable (Elevation—*Elev*, Solar Radiance—*Rad*, Julian Date of sampling—*Date*) included in the best performing models for *C*. *marmotae* and *A*. *laevis* are reported in the table.

**Fig 2:**
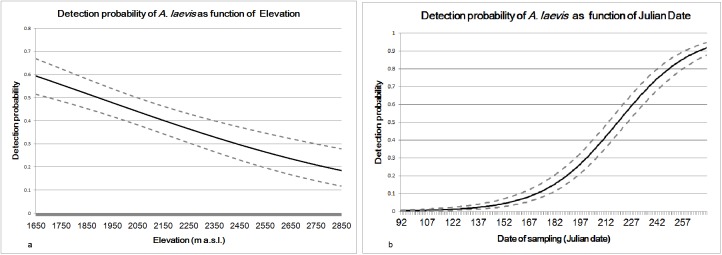
Covariate trend in site occupancy models for *A*. *laevis*. Detection probability (solid line) and confidence intervals (dashed line) of *A*. *laevis* are pictured as function of (2a) Elevation and of Julian date (2b). The represented values of *p* were obtained considering the median value of each covariate within the model [*ψ(*.*)p(Elev+Date)*].

Like *C*. *marmotae*, *A*. *laevis* presented an occupancy of 100% (*ψ* = 1). Site and sampling-specific covariates (solar radiance, elevation, and sampling date) were used to model how parasite detectability (*p*), used as a proxy of abundance, varies across time and space. The best performing model (AIC = 471.35) (Table 3) includes elevation and sampling date [*ψ (*.*)p(Elev+Date)*] (Table 4). Elevation is negatively related to detection probability (Fig 2A) while Julian date has a positive relationship with detection probability (Fig 2B).

## Discussion

Living at high elevations has substantial costs for Alpine marmots: a shorter feeding season influences over-winter survival and longer snow cover in spring affects the reproductive success of female marmots by limiting food resources at the beginning of the active season [18, 49,50]. Each family must have within its home-range adequate food resources and hibernacula [19] which both are limiting factors for marmot’s expansion [51]. Elevation, slope, solar exposure, as well as vegetation type and availability period, type of soil and snow cover permanence influence, to different extents, Alpine marmot’s habitat preferences [23,51]. Marmots at lower elevations are subjected to higher ambient temperatures, which can represent a major cause of stress [52]. On the opposite, high altitude habitats offer colder temperatures but also a shorter vegetative period due to longer snow cover at the end of the winter. Within this complex mosaic of influencing factors, we investigated the potential role of parasitism in constraining the altitudinal niche of Alpine marmots. We hypothesized that less favorable environmental conditions like those that marmots face when living at higher elevations (within their altitudinal range of presence), might be partially compensated by a lower risk of infection with gastrointestinal macro-parasites. By using site-occupancy models [37] we were able to assess the probability of detection *p* (as a predictor of abundance [39]) of *C*. *marmotae* oncospheres and *A*. *laevis* eggs during the entire period of activity in relation to elevation and solar radiance in Alpine marmots. Elevation was a predictor of both *C*. *marmotae* and *A*. *laevis* abundance, and our models suggest that marmot families living at higher elevations have significantly less *C*. *marmotae* and *A*. *laevis* abundances, reflecting a lower risk of infection. A given environment can become less disadvantageous for a species if the same disadvantageous conditions, with the necessary species-specific constraints, apply also to its hosted parasites during their external environmental phase. The effects of elevation on parasites are multiple but mostly referring to the temperature gradient [53, 54, 55]. Temperature is inversely related to elevation and elevation differences can cause significant changes in temperature over short geographic distances [56]. Embryonation rate of Ascarid eggs is directly related to temperature, while egg recovery and survival are favored by winter climatic conditions [57, 58]. Temperature-dependent dynamics of Ascarids can explain the low recorded abundance of *A*. *laevis* at high elevation sites but also their environmental persistence despite harsh winter temperatures. Temperature affects also embryonation of cysticercoids in Oribatid mites as developmental duration is inversely related to temperature [59]. Solar radiance was negatively related to the detection probability of *C*. *marmotae*. The effect of solar exposure on the abundance of *C*. *marmotae* is possibly indirect as it is known that desiccation due to solar exposure and high evaporation rates affects negatively Oribatid abundance [60]. For *A*. *laevis* instead, solar radiance did not appear to be a significant predictor of abundance suggesting resistance of Ascarid eggs to solar ultraviolet radiation as also demonstrated under simulated solar disinfection conditions for parasites of the same genus [61].

For both parasite species, detection probability was positively related to the date of sampling. In the specific case of *A*. *laevis*, this is reflected in the estimate of coprological prevalence which reaches its maximum value in September, towards the end of the active season, as found also by Bassano et al. [24] in the same study area. Coprological prevalence of *C*. *marmotae* instead peaked in July reaching P = 61.90%. We first detected *A*. *laevis* in June, about 70 days after the emergence from hibernation. Our data fall in between the prepatent period of 90 days estimated by Babero [62] and of about 40 days as reported by Callait [63]. Such an extended and variable prepatent period is consistent with two overwintering strategies previously proposed for *A*. *laevis*: larvated eggs as form of external resistance and/or the persistence of migrating larvae in the tissues of marmots during hibernation [24, 27]. There is no definite evidence of successful overwintering of migrating larvae, but studies carried out on golden hamsters (*Mesocricetus auratus*) demonstrated that lowered body temperature in the host inhibits *Trichinella spiralis* development without resulting in the elimination of infection [64, 65]. As for *C*. *marmotae* overwintering might be either realized in the intermediate hosts in the form of infective cysticercoids or by persistence in the hibernating animal [24, 27]. The scolex of *Hymenolepis citelli*, for example, has been recovered from hibernating *Spermophilus tridecemlineatus* [66]. Our results, as those reported by Bassano et al. [24] and Callait and Gauthier [27] suggest that the emission of oncospheres starts in June, a prepatent period that favors the first hypothesis, namely the maintenance of *C*. *marmotae*, in the form of cysticercoid in the intermediate hosts.

The high adaptation of Alpine marmots to their environment corresponds to an extreme specialization of its parasites *C*. *marmotae* and *A*. *laevis*, which are reported in Alpine marmots together with *C*. *alpina*, with high frequency and abundance in studies based on necropsy [63]. We did not detect any egg of *C*. *alpina* but as confirmed previously [24, 27, 63, 67], copromicroscopic analysis is highly unsuitable for this parasite due to its short mating period and to the marked disproportion between sexes during the active season [27]. Coccidia of the genus *Eimeria* were the only other parasites species detected.

## Conclusions

The present work was aimed to investigate from an ecological point of view, the probability of infection of Alpine marmots by two of its most common and abundant parasites (*A*. *laevis* and *C*. *marmotae*) along an altitudinal gradient. Parasitism can affect hosts both at population level with an indirect demographic effect [6] and directly by influencing individual animal behavior [68, 69]. The hypothesis of a trade-off between the cost of living at higher elevations and the lower risk of parasite infection was supported by site occupancy models developed on presence data of *A*. *laevis* and *C*. *marmotae* detected by coprological examinations. The insights on the possible trade-off between habitat-suitability and lower parasite load that emerged from this study will be useful to further investigate patterns of habitat selection and the effects of parasite infection on Alpine marmot fitness and life-history. Habitat preferences of alpine marmots have been evaluated in regard to a variety of environmental factors that to different extents influence marmot’s habitat preferences [70]. Within the complex mosaic of influencing factors, it has been demonstrated that certain elements are preponderant in given situations, and others under different circumstances [18]. Further research is needed to assess how parasitism can affect individual fitness and thus have a demographic effect on marmot communities at lower elevations.

The existence of a threshold value in parasite load will also need further investigation. To our knowledge, only few studies have investigated, under natural settings, the constraints that parasite infections can make on the distributional niche of a wild species [14], especially in birds in the case of malaria and vector-borne diseases [12, 13]. Site-occupancy models have been used previously to investigate the prevalence of ecto-parasitic vectors on hosts [71], the detection of pathogens through the histological examination of tissue samples [72] and PCR diagnosis of malaria in blood samples [73]. In this study instead we exploit, for the first time, site occupancy models to formally overcome the issue of imperfect detection in studies based on the detection of parasite life stages in fecal samples. The inconstant shedding and thus detection, of parasitic developmental stages (eggs, oncospheres, or gravid proglottids) is an intrinsic limit of copromicroscopic diagnosis [34]. We thus propose the use of site occupancy models in any eco-parasitological study where the detection probability is lower than one.
